# Transcriptomic Reprogramming in Leaves During Floral Bud Morphogenesis in Blueberry

**DOI:** 10.3390/genes17030317

**Published:** 2026-03-14

**Authors:** Xingyu Lu, Dongyu Sun, Yiyan Yang, Ya Shen, Qin Yang, Biyan Zhou

**Affiliations:** 1 Provincial Famous Teacher Yang Qin Studio/Guizhou Key Laboratory of Molecular Breeding for Characteristic Horticultural Crops, College of Life and Health Science, Kaili University, Kaili 556011, China; luxingyu90@163.com (X.L.); shenyaella@163.com (Y.S.); 2 College of Horticulture, South China Agricultural University, Guangzhou 510642, China; dy-sun@outlook.com (D.S.); 13202002376@163.com (Y.Y.)

**Keywords:** blueberry, floral bud morphogenesis, leaf transcriptome, WGCNA, regulatory network

## Abstract

**Background/Objectives**: Floral bud morphogenesis is a critical developmental process determining yield potential in blueberry, yet the molecular regulatory mechanisms in leaves during this phase remain poorly understood. **Methods:** In this study, we employed a time-series transcriptomic approach to investigate leaf gene expression dynamics during floral bud morphogenesis in rabbiteye blueberry. Leaves were sampled at six time points spanning the critical developmental window from the cessation of summer shoot growth to bud swell and dormancy onset. **Results:** RNA-seq analysis generated 121.68 Gb of clean data, and weighted gene co-expression network analysis (WGCNA) identified four stage-specific modules (brown, red, blue, turquoise) significantly associated with distinct morphogenetic phases. The brown module (0–6W) was enriched in photosynthesis and hormone signaling pathways, while the red (9W) and blue (12W) modules featured protein processing, stress and hormone signaling, and carbohydrate metabolism. The turquoise module (15W) was dominated by carbon metabolism and flavonoid biosynthesis genes. Key flowering-related genes exhibited dynamic expression patterns: *FT* was specifically upregulated at the late stage (15W), *AP2* genes peaked at mid-stage (9–12W), and *COL9* showed early high expression (0–3W). Hormone-related gene analysis revealed extensive involvement of multiple pathways, with brassinosteroid (BR) signaling comprising the largest number of genes (101). Co-expression networks further identified hub genes, including *FT*, *COL9*, *AP2*, *ERF1*, *SR160*, *LOX3-1*, and transcription factor genes like *MYB-related*, as potential central regulators. **Conclusions:** Our findings demonstrate that blueberry leaves undergo a phased functional transition from a photosynthetic source to a hub for signal integration and metabolic support during floral bud morphogenesis, actively contributing to reproductive development through systemic signaling. This study provides novel insights into flowering regulation in woody perennials and establishes a foundation for marker-assisted breeding and cropping season management in blueberry.

## 1. Introduction

Blueberry (*Vaccinium* spp.), a shrub belonging to the Ericaceae family, is native to North America. The fruits are rich in anthocyanins and other antioxidants, conferring health benefits such as improved vision and enhanced immunity. With its high economic value, blueberry is now widely cultivated across the globe [1]. Floral bud differentiation is a critical physiological process for yield formation, directly determining flowering quality and fruit set in the following year. The floral bud morphogenesis phase, which encompasses the physiological differentiation and early morphological differentiation of flower buds, represents the pivotal stage when blueberry transitions from vegetative to reproductive growth and completes the differentiation of floral organ primordia. This phase constitutes a central hub in the plant’s annual growth cycle. Similar to most temperate woody fruit trees, floral bud differentiation in blueberry initiates during the preceding growing season, with the differentiation of floral organ primordia being completed during the floral bud morphogenesis stage, thereby establishing the foundation for the subsequent spring flowering and fruiting [2,3]. Therefore, deciphering the molecular regulatory mechanisms underlying this phase is of great significance for optimizing cropping season regulation and achieving high-yield cultivation in blueberry.

Floral bud differentiation is a complex, programmed process coordinately regulated by internal genetic factors and external environmental signals. In the model plant *Arabidopsis*, various flowering regulatory pathways, including vernalization, photoperiod, gibberellin, aging, autonomous, and ambient temperature pathways, ultimately converge on floral pathway integrators such as *FT* (*FLOWERING LOCUS T*) and *SOC1* (*SUPPRESSOR OF OVEREXPRESSION OF CONSTANS 1*). These integrators in turn regulate floral meristem identity genes, thereby determining flowering time [4,5]. Leaves serve as the primary organs for perceiving environmental signals such as light and temperature. Florigenic substances synthesized in leaves, notably the FT protein, are transported via the phloem to the shoot apical meristem, initiating the floral transition [6,7]. In perennial woody species, however, the regulation of this leaf-derived signaling pathway is often more intricate, integrating additional factors such as photoperiod signals, dormancy cycles, chilling requirements, and age-related pathways [8]. For instance, studies in fruit trees like apple (*MdFT*), citrus (*CiFT*), and kiwifruit (*AcFT*) have demonstrated that *FT* homologs not only integrate the environmental signals but also coordinate flowering with perennial growth habits [9,10,11]. Furthermore, hormones act as crucial chemical messengers mediating communication between leaves and the shoot apex. Complex hormone signaling networks operating within leaves, involving gibberellins (GA), abscisic acid (ABA), cytokinins (CTK), and others, are also deeply involved in the integration and transmission of floral inductive signals [12,13].

In recent years, research on reproductive growth in blueberry has primarily focused on fruit development and quality formation. Particular attention has been paid to the molecular mechanisms underlying anthocyanin accumulation during fruit ripening, the analysis of fruit nutritional composition and functional evaluation, as well as postharvest storage and preservation technologies [14,15]. Regarding the molecular regulation of flowering in blueberry, the research group led by Guo-Qing Song at Michigan State University has conducted systematic investigations. These studies have explored the regulatory mechanisms of flowering involving environmental and endogenous factors such as chilling accumulation, photoperiod response, and hormone signals, and have included functional validation of key flowering-related genes (e.g., *FT*, *SOC1*, *SVP*) [16,17,18,19]. Additionally, the role of sugar signals as both energy sources and signaling molecules in blueberry flowering has been reported [20,21], revealing a close association between carbon metabolism and flowering regulation. However, studies on the temporal dynamics of genome-wide gene expression, core regulatory networks, and their coordination with multi-hormone signaling in leaves specifically during the floral bud morphogenesis phase in blueberry remain relatively scarce.

Guizhou Province, located in southwestern China, is a major blueberry production region due to its unique climatic conditions [22]. The rabbiteye blueberry cultivar ‘Brightwell’ is predominantly grown in open-field conditions in this area, exhibiting strong adaptability and high productivity. In this study, we employed a time-series transcriptomic approach to investigate the molecular mechanisms underlying floral bud morphogenesis in leaves of six-year-old ‘Brightwell’ plants. Leaf samples were collected at six time points spanning the critical developmental window from the cessation of summer shoot growth to bud swell and the onset of dormancy. RNA-seq coupled with weighted gene co-expression network analysis (WGCNA) was used to identify core gene modules associated with distinct morphogenetic stages. Key genes involved in flowering regulation, hormone signal transduction, and hormone biosynthesis/metabolism, as well as transcription factor-encoding genes within these modules were systematically explored to construct gene co-expression regulatory networks. By elucidating the leaf transcriptomic reprogramming that accompanies floral bud morphogenesis, this study provides new insights into the flowering regulatory mechanisms of woody perennials. It also establishes a foundation for marker-assisted breeding and cropping season management in blueberry.

## 2. Materials and Methods

### 2.1. Plant Materials and Sampling

The experiment was conducted using six-year-old, open-field cultivated plants of the rabbiteye blueberry cultivar ‘Brightwell’. Twelve healthy plants with uniform growth vigor (approximately 1.5–2.0 m in height) were selected. The plants were grown in raised beds (25 m length × 1 m width × 0.8 m height) filled with a substrate mixture of leaf mold, pine needle soil, and peat moss (4:3:2, *v*/*v*/*v*). The experimental site was located at the Excellent Agri-Forestry Talents Training Practice Base of Kaili University (26°31′5.92″ N, 107°51′23.18″ E).

Leaf samples were collected from the 4th to 6th nodes of vigorous one-year-old shoots (the primary flowering and fruiting shoots) during the floral bud morphogenesis phase. Sampling was performed at six time points at three-week intervals from 14 July to 27 October 2023 (designated as 0, 3, 6, 9, 12, and 15 weeks; 0W, 3W, 6W, 9W, 12W, 15W). During the experimental period, the average daily maximum temperature in the experimental plots decreased from approximately 30 °C to 20 °C, while the average daily minimum temperature dropped from around 20 °C to 10 °C. The initiation of sampling (0W) was determined by the cessation of summer shoot growth after harvest, marked by the appearance of “black tips” (aborted, blackened apical leaves on new shoots). Sampling continued until floral bud swell and the onset of dormancy were observed (15W). At each sampling event, two leaves were collected from each of the four cardinal directions (east, south, west, north) per plant. Leaves from four plants were pooled to constitute one biological replicate. Three biological replicates per time point, yielding a total of 18 samples. All collected samples were immediately frozen in liquid nitrogen and stored at −80 °C for subsequent analysis.

### 2.2. RNA Extraction, Library Construction, and Data Analysis

Total RNA was extracted by using a polysaccharide- and polyphenol-rich plant total RNA extraction kit (DP441, Tiangen Biotech, Beijing, China), following the manufacturer’s instructions. RNA concentration and purity were determined using a NanoDrop 2000 spectrophotometer (Thermo Scientific, Waltham, MA, USA), and RNA integrity was assessed using an Agilent Bioanalyzer 2100 system with the RNA Nano 6000 assay kit (Agilent Technologies, Santa Clara, CA, USA). For each sample, 1 μg of total RNA was used as input material for library construction with the Hieff NGS Ultima Dual-mode mRNA Library Prep Kit for Illumina (Yeasen Biotechnology, Shanghai, China). The main steps included: mRNA enrichment using oligo(dT)-attached magnetic beads, followed by fragmentation with Fragmentation Buffer; first-strand cDNA synthesis using the fragmented mRNA as template, followed by second-strand cDNA synthesis; end repair of double-stranded cDNA to generate blunt ends using exonuclease and polymerase activities, followed by A-tailing and adapter ligation; fragment size selection and purification using AMPure XP beads (Beckman Coulter, Pasadena, CA, USA); and final library amplification via PCR enrichment. Library concentration and insert fragment size were assessed using a Qubit 3.0 fluorometer and a Qsep400 high-throughput analysis system, respectively. Sequencing was performed on the Illumina NovaSeq platform (Illumina, San Diego, CA, USA). Raw data from the 18 libraries have been deposited in the China National Genomics Data Center (https://ngdc.cncb.ac.cn/) under BioProject accession number PRJCA056783.

Raw reads were processed to remove adapters, low-quality sequences (reads with poly-N content greater than 10%, or with more than 50% of bases having a quality score Q ≤ 10), and rRNA sequences to obtain clean data. Clean reads were aligned to the blueberry reference genome (https://www.vaccinium.org/analysis/49, accessed on 30 November 2025) [23] using HISAT2 [24]. Correlation among biological replicates and sample dispersion were assessed through inter-sample correlation analysis and principal component analysis. Gene functional annotation was performed using the BMKCloud platform (https://international.biocloud.net/zh/software/tools/detail/small/308, accessed on 8 December 2025) against the Nr (NCBI non-redundant protein sequences), Pfam, Swiss-Prot, GO (Gene Ontology), KEGG (Kyoto Encyclopedia of Genes and Genomes), and COG (Cluster of Orthologous Groups of proteins) protein databases. Gene expression levels were quantified as fragments per kilobase of transcript per million mapped reads (FPKM) using StringTie (version 2.2.3) with the maximum flow algorithm. Differential expression analysis was conducted using the R (version 4.4.2) package DESeq2 [25], with genes satisfying |fold change| ≥ 2 and FDR < 0.01 considered as differentially expressed genes (DEGs).

### 2.3. Weighted Gene Co-Expression Network Analysis (WGCNA) and Screening of Core Module Genes

Co-expression network analysis was performed on the identified DEGs using the WGCNA tool on the BMKCloud platform (https://international.biocloud.net/zh/software/tools/detail/small/8a8300b253cf73e70153d16368250f32, accessed on 22 December 2025). Parameters were set as follows: genes with FPKM > 0 were included in the analysis; the softPower was set to 0 (default values, automatically undergo soft thresholding based on the model); the similarity threshold for module merging was set to 0.5; and the minimum number of genes per module was set to 20. Genes within each obtained module were subjected to KEGG functional annotation and transcription factor identification. Subsequently, we focused on screening for genes and transcription factors within core modules that are associated with flowering regulation, hormone signal transduction, and hormone biosynthesis and metabolism. This screening process was primarily guided by KEGG annotation results, with reference to the multi-species flowering gene regulatory network database (http://pfgd.bio2db.com/) [26] and the multi-species plant hormone biosynthesis, metabolism, signal transduction, and transport gene database (http://phgd.bio2db.com/) [27]. Finally, heatmaps illustrating the expression patterns of these key genes were generated using TBtools software (version 2.154) [28].

### 2.4. Construction of Co-Expression Regulatory Network

Gene co-expression networks for core modules were visualized using Cytoscape (version 3.9.0). The networks prominently feature flowering-related genes, hormone signal transduction-related genes, hormone biosynthesis/metabolism-related genes, and transcription factor genes. Due to the relatively small number of genes in the brown and red modules, no threshold filtering was applied. For the blue and turquoise modules, weight thresholds of 0.65 and 0.55, respectively, were used for network construction. In the visualized networks, node size is proportional to its connectivity (degree), with larger nodes indicating a higher number of interactions with other genes within the network. Edge color represents the connection weight between two genes, with a gradient from light yellow to pink indicating increasing weight values. Hub genes were identified as those with the highest Degree (Weight) values calculated by the CytoNCA app within Cytoscape, which integrates both the degree and the weight value.

### 2.5. Quantitative Real-Time PCR (qPCR) Validation

Total RNA was extracted from reserved sample aliquots following the method described in Section 2.2. For each sample, 1 μg of total RNA was reverse transcribed into cDNA using the Hifair^®^ II 1st Strand cDNA Synthesis Kit (with gDNA digester plus) (Yeasen Biotechnology, Shanghai, China) according to the manufacturer’s protocol. Gene-specific primers were designed using Primer Premier 5.0 software. The *ACTIN* gene [19] was used as an internal reference control. Primer sequences are listed in Supplementary Table S1. qPCR reactions were performed on a CFX96 Real-Time PCR System (Bio-Rad, Hercules, CA, USA) using Hieff^®^ qPCR SYBR Green Master Mix (No Rox, Yeasen Biotechnology, Shanghai, China). The thermal cycling program was as follows: 95 °C for 5 min (initial denaturation); 40 cycles of 95 °C for 5 s, 58 °C for 20 s, and 72 °C for 20 s. Three technical replicates were performed for each sample. Relative gene expression levels were calculated using the 2^−ΔΔCT^ method [29]. Statistical significance of qPCR results was analyzed using the SPSS software (version 20.0) by one-way analysis of variance (ANOVA) followed by Duncan’s multiple range test (*p* < 0.05).

## 3. Results

### 3.1. Quality Assessment of Transcriptome Sequencing Data

Eighteen cDNA libraries were constructed from blueberry leaves sampled during the floral bud morphogenesis phase (six time points: 0, 3, 6, 9, 12, and 15 weeks, with three biological replicates per time point) and sequenced using high-throughput technology. After removing adapters, filtering low-quality reads, and eliminating rRNA sequences, a total of 121.68 Gb of Clean Data were obtained. The Clean Data volume for each library ranged from 5.91 × 10^9^ to 8.42 × 10^9^ bp, with clean reads numbering between 3.97 × 10^7^ and 5.68 × 10^7^. The mapping rates of reads from all samples to the reference genome ranged from 84.85% to 90.16%, indicating high sequencing data quality and alignment efficiency (Table 1).

As shown in Supplementary Figure S1, both sample correlation analysis and principal component analysis (PCA) demonstrated good consistency among the three biological replicates within each time point, reflecting high similarity between replicates. Furthermore, a clear separation was observed among three sample groups: 0W/3W/6W, 9W/12W, and 15W. Notably, the 15W samples exhibited the most pronounced dispersion, suggesting a substantial shift in the gene expression profile at this time point.

### 3.2. Identification of Differentially Expressed Genes

**Figure 1:**
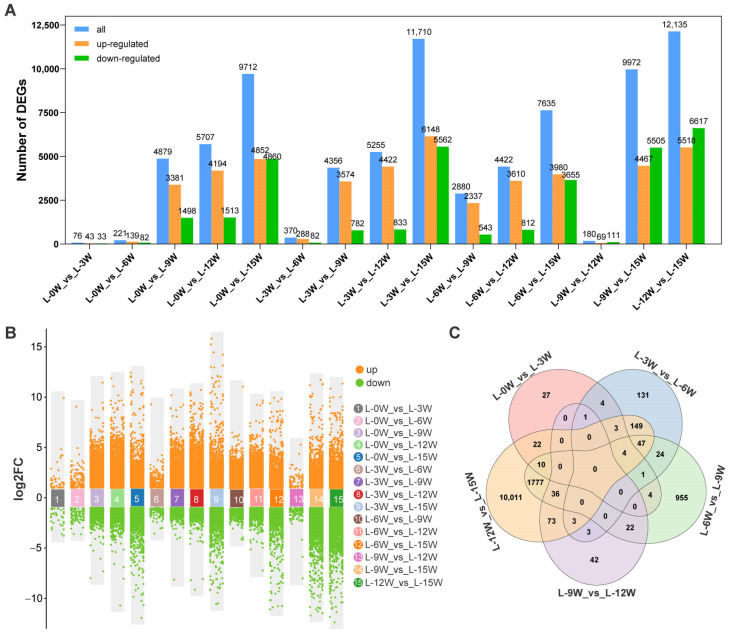
Identification of differentially expressed genes (DEGs) in blueberry leaves during floral bud morphogenesis. (**A**) Bar plot showing the number of DEGs (log2FC ≥ 2, FDR < 0.01) identified in each comparison. (**B**) Volcano plots visualizing DEGs for each pairwise comparison. (**C**) Venn diagram of the DEGs from adjacent time point comparisons.

Differential expression analysis identified a total of 22,463 differentially expressed genes (DEGs). As illustrated in Figure 1A,B, the number of DEGs between adjacent early time points was relatively low: 76 between 0W and 3W, 221 between 3W and 6W, 370 between 6W and 9W, and 180 between 9W and 12W. In contrast, comparisons between each of the earlier time points (0W, 3W, 6W, 9W, 12W) and 15W revealed dramatically increased numbers of DEGs, totaling 9712, 11710, 7635, 9972, and 12135, respectively. This result indicates that the gene expression pattern at 15W (corresponding to the stage following rapid bud swell and the onset of dormancy) is markedly different from that of the preceding five time points. Based on this data profile and the observed developmental progression, DEGs from all adjacent time point comparisons (0W vs. 3W, 3W vs. 6W, 6W vs. 9W, 9W vs. 12W, 12W vs. 15W) were combined into a union set, yielding a total of 13,349 DEGs for weighted gene co-expression network analysis (WGCNA) (Figure 1C).

### 3.3. Weighted Gene Co-Expression Network Analysis and Functional Enrichment of Core Modules

**Figure 2:**
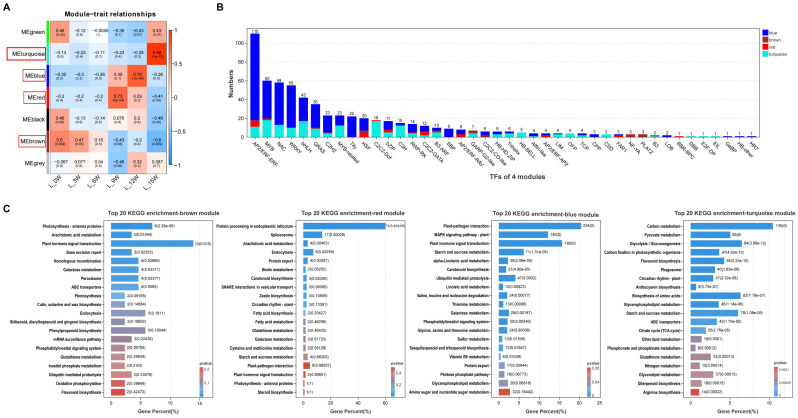
Weighted gene co-expression network analysis (WGCNA), transcription factor identification, and KEGG enrichment analysis of core modules. (**A**) Heatmap displaying the correlation between modules and sampling time points. (**B**) Number of transcription factors identified in each of the four core modules. (**C**) KEGG functional enrichment analysis of genes within the four core modules (numbers on the right side of the bars indicate the gene count and *p*-value for each enriched pathway).

A weighted gene co-expression network was constructed using the 13,349 DEGs, resulting in the identification of seven distinct co-expression modules (Figure 2A). Among these, four modules (brown, red, blue, and turquoise) exhibited significant correlations with specific sampling time points and were therefore designated as core modules. The module eigengene (ME) for the brown module (MEbrown) showed high expression levels at 0W, 3W, and 6W, but low expression at 9W, 12W, and 15W. Genes in this module were significantly enriched in pathways related to photosynthesis, arachidonic acid metabolism, and plant hormone signal transduction. The red module’s eigengene peaked at 9W, with its genes enriched in protein processing in the endoplasmic reticulum, the spliceosome, and arachidonic acid metabolism. The blue module exhibited highest expression at 12W and was enriched in plant–pathogen interaction, the MAPK signaling pathway, plant hormone signal transduction, starch and sucrose metabolism, and alpha-Linolenic acid metabolism. The turquoise module showed highest expression at 15W, with genes enriched in carbon metabolism, pyruvate metabolism, glycolysis/gluconeogenesis, carbon fixation in photosynthetic organisms, and flavonoid biosynthesis (Figure 2A,C). Furthermore, transcription factor (TF) prediction within these four core modules identified 627 TFs belonging to 42 families, the majority of which were distributed within the blue module (Figure 2B).

### 3.4. Screening and Expression Pattern Analysis of Key Genes Within Core Modules

From the combined set of 6916 genes comprising the four core modules (brown, red, blue, turquoise), we identified 16 flowering-related genes, 262 genes associated with hormone signal transduction, and 258 genes involved in hormone biosynthesis and metabolism.

**Figure 3:**
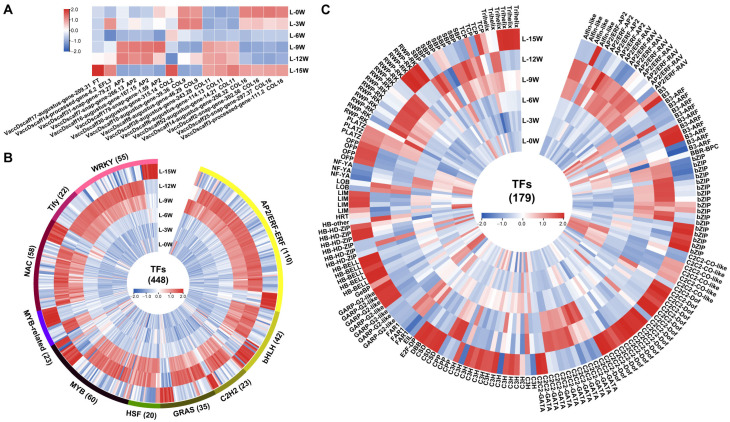
Expression heatmaps of transcription factors and flowering-related genes within core modules. (**A**) Heatmap of flowering-related genes. (**B**) Heatmap of genes from the top 10 most abundant transcription factor families. (**C**) Heatmap of genes from other transcription factor families.

The flowering-related genes displayed diverse expression patterns (Figure 3A). The key florigen gene *FT* was significantly upregulated at 15W. Four *AP2* (*APETALA2*) genes were predominantly expressed at 9W and 12W, whereas two *COL9* (*CONSTANS-LIKE 9*) genes showed high expression at 0W and 3W. Three *COL11* genes were highly expressed at 9W, 12W, and 15W, and four *COL16* genes exhibited high expression at 0W, 3W, and 15W. Additionally, a *COL2* gene was highly expressed at 15W, and an *EFL2* gene showed two expression peaks at 3W and 15W.

Analysis of transcription factors revealed that the AP2/ERF-ERF, MYB, NAC, WRKY, bHLH, GRAS, MYB-related, C2H2, Tify, and HSF families were the ten most abundant (Figure 3B,C). The majority of TF-encoding genes were highly expressed at 9W, 12W, and 15W, with particularly high numbers observed at 9W and 12W. The bHLH and MYB-related families showed a relatively balanced distribution of genes highly expressed at 9W/12W versus those highly expressed at 15W. Families such as C2C2-GATA, bZIP, RWP-RK, SBP, and AP2/ERF-RAV were predominantly expressed at 9W and 12W, whereas families including C2C2-Dof, C3H, Trihelix, and HB-BELL were mainly expressed at 15W.

**Figure 4:**
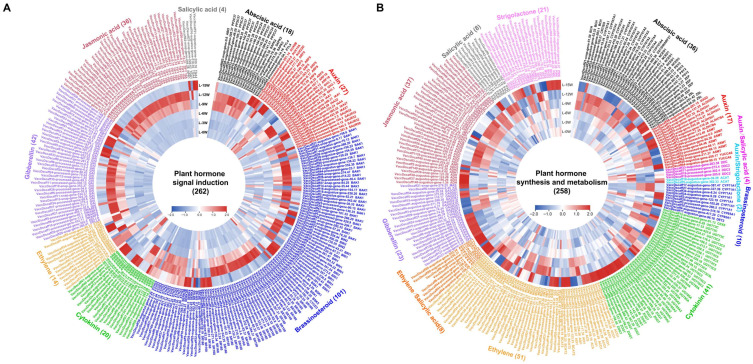
Expression heatmaps of genes involved in hormone signal transduction (**A**), hormone synthesis and metabolism (**B**) within core modules.

Genes involved in hormone signal transduction spanned eight major pathways: abscisic acid (ABA), auxin (IAA), brassinosteroid (BR), cytokinin (CTK), ethylene (ETH), gibberellin (GA), jasmonic acid (JA), and salicylic acid (SA) (Figure 4A). The BR signaling pathway contained the largest number of genes (101), most of which were highly expressed at 9W/12W or 15W. JA signaling genes (36) were uniformly highly expressed at 9W and 12W, while all 42 GA signaling genes were upregulated at 9W/12W or 15W. In contrast, in the ABA (18 genes), IAA (27 genes), CTK (20 genes), ETH (14 genes), and SA (4 genes) pathways, only a few genes showed early expression, with the majority also being upregulated during the middle to late stages.

Similarly, most genes involved in hormone biosynthesis and metabolism were predominantly up-regulated during the middle to late stages (9W–15W) of floral bud morphogenesis (Figure 4B). ETH biosynthesis and metabolism involved the largest number of genes (51), followed by CTK (41 genes), JA (37 genes), ABA (36 genes), GA (23 genes), and SL (21 genes) pathways. Smaller gene sets were associated with IAA (17 genes), BR (10 genes), and SA (8 genes) biosynthesis and metabolism. Relatively few hormone-related genes were highly expressed during the early 0–6W period.

### 3.5. Validation by Quantitative Real-Time PCR (qRT-PCR)

**Figure 5:**
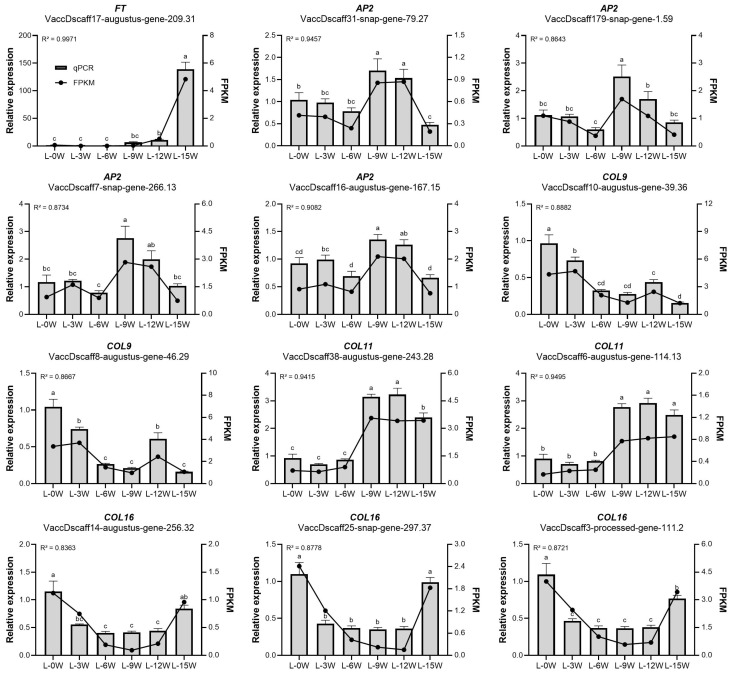
Validation of selected genes by quantitative real-time PCR (qRT-PCR). The left *y*-axis corresponds to the relative expression level determined by qRT-PCR, and the right *y*-axis corresponds to the FPKM value from RNA-Seq. The R^2^ value in the upper-left corner of each panel represents the correlation coefficient between the two datasets. Different lowercase letters indicate significant differences among different time points (*p* < 0.05, Duncan’s multiple range test).

Twelve genes were randomly selected from the four core modules for validation by quantitative real-time PCR (qRT-PCR). As shown in Figure 5, the expression trends obtained from qRT-PCR were generally consistent with the FPKM values derived from RNA-Seq. Correlation analysis between the qRT-PCR and RNA-Seq results for each validated gene yielded correlation coefficients (R^2^) greater than 0.8, indicating a high degree of concordance and confirming the reliability of the transcriptome sequencing data.

### 3.6. Construction of Gene Co-Expression Networks for Core Modules

**Figure 6:**
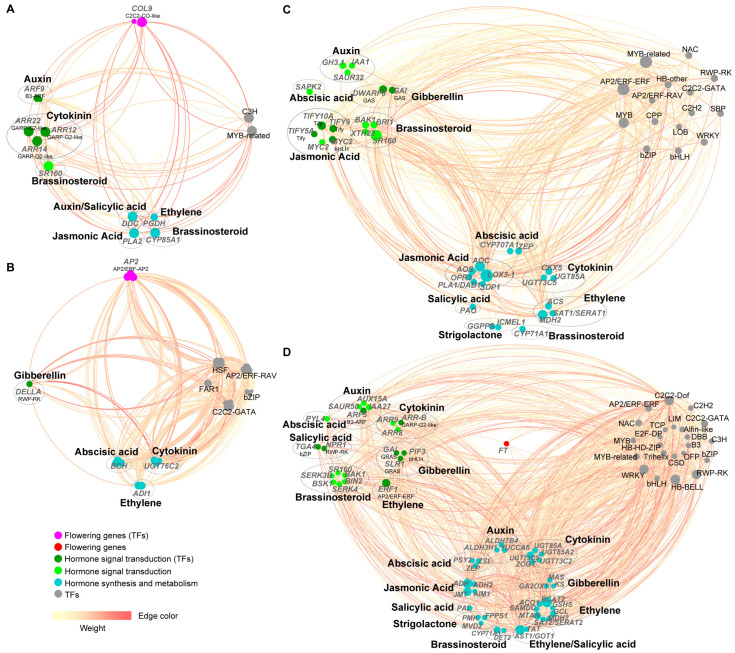
Visualization of gene co-expression networks for core modules. (**A**) Co-expression network for the brown module highlighting flowering-related genes, hormone signal transduction-related genes, hormone biosynthesis/metabolism-related genes, and transcription factors. (**B**) Co-expression network for the red module highlighting the four gene categories. (**C**) Co-expression network for the blue module highlighting the four gene categories. (**D**) Co-expression network for the turquoise module highlighting the four gene categories.

To further investigate the regulatory relationships among key genes within the core modules, gene co-expression networks were constructed separately for the brown, red, blue, and turquoise modules. These networks specifically highlight the interconnections among flowering-related genes, hormone signal transduction-related genes, hormone biosynthesis/metabolism-related genes, and transcription factors (Figure 6).

As shown in Figure 6A (brown module), the flowering-related gene connected to other genes within this module was *COL9*. Hormone signal transduction-related genes included *ARF9* (IAA), *ARR12/14/22* (CTK), and *SR160* (BR). Hormone biosynthesis/metabolism-related genes comprised *DDC* (IAA and SA), *PGDH* (ETH), *CYP85A1* (BR), and *PLA2* (JA). The predominant transcription factors belonged to the C3H and MYB-related families. Based on integrated connectivity (degree) and weight values, the hub genes representing the four categories within this module were identified as *COL9* (VaccDscaff10-augustus-gene-39.36), *ARR12* (VaccDscaff43-processed-gene-102.10), *PLA2* (*phospholipase A2*, VaccDscaff40-augustus-gene-211.24), and a *MYB-related* gene (VaccDscaff24-augustus-gene-224.16).

In the red module (Figure 6B), flowering-related genes connected within the network was *AP2*. Hormone signal transduction-related genes were represented by *DELLA* (GA). Hormone biosynthesis/metabolism-related genes included *BCH* (ABA), *UGT76C2* (CTK), and *ADI1* (ETH). Transcription factors encompassed HSF, AP2/ERF-RAV, bZIP, C2C2-GATA, and FAR1 families. Hub genes for the four categories were *AP2* (VaccDscaff179-snap-gene-1.59), *DELLA* (VaccDscaff20-snap-gene-360.41), *BCH* (*beta-carotene hydroxylase*, VaccDscaff21-snap-gene-51.50), and an *HSF* gene (VaccDscaff3-augustus-gene-415.25).

Within the blue module (Figure 6C), connected hormone signal transduction-related genes spanned multiple pathways, including IAA (e.g., *GH3.1*), GA (e.g., *DWARF8*), BR (e.g., *BAK1*), JA (e.g., *MYC2*), and ABA (*SAPK2*). Hormone biosynthesis/metabolism-related genes were involved in CTK (e.g., *CKX5*), ETH (e.g., *ACS*), ABA (e.g., *CYP707A1*), BR (*CYP71A1*), SA (*PAO*), JA (e.g., *LOX3*-1), and SL (e.g., *GGPPS*) pathways. A rich diversity of transcription factor families was observed, including MYB, MYB-related, AP2/ERF-ERF. Hub genes representing the four categories were *SR160* (*systemin receptor SR160*, VaccDscaff16-augustus-gene-92.33), *LOX3-1* (*linoleate 13S-lipoxygenase 3-1*, VaccDscaff9-augustus-gene-407.29), and a *MYB-related* gene (VaccDscaff27-augustus-gene-321.25).

In the turquoise module (Figure 6D), the connected flowering-related gene was *FT* (VaccDscaff17-augustus-gene-209.31). Hormone signal transduction-related genes spanned IAA (e.g., *ARF5*), GA (e.g., *GAI*), CTK (e.g., *ARR8*), BR (e.g., *BRI1*), ETH (*ERF1*), ABA (*PYL4*), and SA (e.g., *NPR1*) pathways. Hormone biosynthesis/metabolism-related genes were involved in IAA (e.g., *YUCCA6*), GA (e.g., *GA2OX1*), CTK (e.g., *UGT85A*), ABA (e.g., *ZEP*), ETH (e.g., *ACO1*), BR (e.g., *CYP71A1*), SA (*PAL*), JA (e.g., *ADH*), and SL (e.g., *PMK*) pathways. Transcription factor families included C2C2-Dof, AP2/ERF-ERF, HB-BELL. Hub genes representing the four categories were *FT* (VaccDscaff17-augustus-gene-209.31), *ERF1* (ethylene-responsive transcription factor 1, VaccDscaff15-processed-gene-360.12), *BCAT2* (branched-chain-amino-acid aminotransferase 2, VaccDscaff41-snap-gene-18.19), and a *HB-BELL* gene (VaccDscaff15-augustus-gene-106.16).

**Figure 7:**
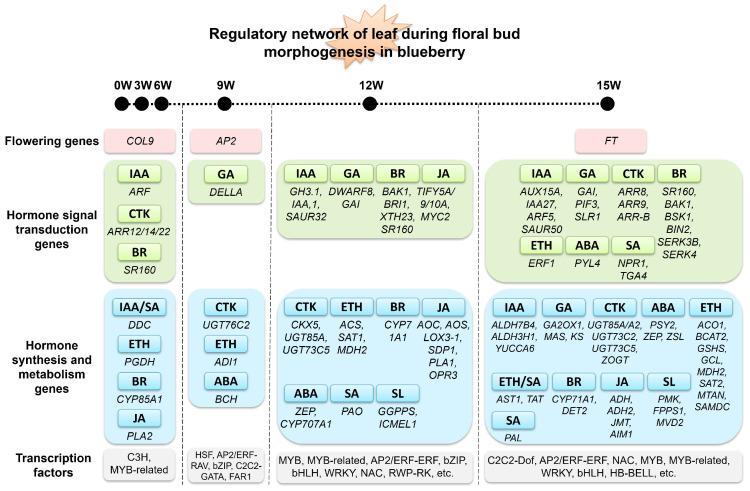
Schematic summary of the regulatory network in blueberry leaves during floral bud morphogenesis.

Synthesizing the co-expression network analyses, distinct temporal regulatory signatures emerged across the floral bud morphogenesis phase (Figure 7). During the early stage (0W, 3W, 6W; corresponding to the brown module), the regulatory network was primarily characterized by the flowering gene *COL9*, genes related to IAA/CTK/BR signal transduction, genes involved in IAA/ETH/BR/JA biosynthesis/metabolism, and transcription factors from the C3H and MYB-related families. At 9W (red module), the network featured the flowering gene *AP2*, GA signal transduction-related genes, genes for CTK/ETH/ABA biosynthesis/metabolism, and transcription factors including HSF, AP2/ERF-RAV, and bZIP. At 12W (blue module), the network involved genes related to IAA/GA/BR/JA signal transduction, genes for CTK/ETH/BR/JA/ABA/SA/SL biosynthesis/metabolism, and a diverse array of transcription factors such as MYB, MYB-related, AP2/ERF-ERF, bZIP, bHLH, WRKY, NAC, and RWP-RK. During the final stage (15W; turquoise module), the network was centered on the flowering gene *FT*, coordinating genes involved in IAA/GA/CTK/BR/ETH/ABA/SA signal transduction, genes for IAA/GA/CTK/ABA/ETH/BR/JA/SA/SL biosynthesis/metabolism, and transcription factors from families including C2C2-Dof, AP2/ERF-ERF, NAC, MYB, MYB-related, WRKY, bHLH, and HB-BELL, collectively constituting the core regulatory network for signal output from leaves at this terminal phase.

## 4. Discussion

As the primary sites of photosynthesis, plant leaves are also crucial for perceiving environmental signals and generating florigenic stimuli. Through transcriptomic analysis of blueberry leaves at six time points during floral bud morphogenesis, this study revealed a distinct phased reprogramming of gene expression patterns. This suggests that leaves undergo a functional transition from a “photosynthetic source” to a hub for “signal integration and metabolic support” during this critical developmental window.

Differential gene expression analysis revealed a dramatic increase in the number of DEGs at the late stage of floral bud morphogenesis (15W) compared to earlier time points (Figure 1), identifying this period as a key node of substantial transcriptomic reprogramming in leaves. Weighted gene co-expression network analysis effectively partitioned the DEGs into seven distinct co-expression modules. Four of these modules (brown, red, blue, and turquoise) showed significant associations with specific phases of floral bud morphogenesis, revealing an orderly, stage-specific transcriptional program in leaves (Figure 2). During the early stage (0–6W), genes in the brown module were significantly enriched in pathways related to photosynthesis and plant hormone signal transduction. This aligns with the leaf’s role during this period in maintaining photosynthetic capacity to provide energy for the plant, while simultaneously beginning to perceive signals such as photoperiod [30,31]. Progressing into the mid-stage (9–12W), the blue and red modules became dominant. Enrichment of pathways, such as plant–pathogen interaction, MAPK signaling, plant hormone signal transduction, RNA processing, and starch and sucrose metabolism, suggests a functional shift in leaves from pure photoassimilation towards signal integration and metabolite accumulation. MAPK cascades in plants are known to participate not only in stress responses but also broadly in developmental regulation [32], while enhanced starch and sucrose metabolism positively contributes to floral bud differentiation in blueberry [21]. At the late stage (15W), the turquoise module became predominant, enriched in pathways including carbon metabolism, pyruvate metabolism, glycolysis/gluconeogenesis, and flavonoid biosynthesis. Enhanced carbon metabolism likely provides sufficient energy for bud swelling, while the accumulation of secondary metabolites like flavonoids may not only enhance the plant’s antioxidant capacity but also play a regulatory role in floral bud differentiation [33].

The temporal expression patterns of flowering-related genes further support the concept of a phased functional transition in leaves. The florigen gene *FT* exhibited a striking, specific upregulation during the middle to late stages of floral bud morphogenesis (12–15W; Figure 3). *FT* encodes a key long-distance mobile signal protein synthesized in leaves that integrates flowering signals and initiates floral bud morphogenesis [6]. Studies in lychee have demonstrated that leaves perceive low temperature, producing flowering signals such as the LcFT1 protein, which is transported via the phloem to the apical bud meristem to induce flowering [34,35]. In blueberry, overexpression of *VcFT* promotes early flowering and partially substitutes for the chilling requirement [16]. Furthermore, grafting non-transgenic blueberry scions onto *VcFT*-overexpressing rootstocks induced early flowering in the scion, providing evidence that VcFT protein synthesized in leaves can be transported in the phloem and function as a systemic signal [18]. Unlike its expression pattern in most annual model plants, where *FT* is primarily expressed during floral induction [36], *FT* homologs in perennial woody species like citrus [37] and lychee [38] maintain high expression during floral organ development. This sustained expression is thought to continuously provide floral signals ensuring proper primordia differentiation. Our results align with these findings, suggesting that *FT* may continue to function during the late stages of floral bud morphogenesis in blueberry.

Several members of the *CONSTANS-LIKE* (*COL*) gene family also displayed dynamic expression patterns. The *CO* gene family is central to the photoperiod pathway, influencing flowering time by regulating *FT* expression [39]. In this study, *COL9* was highly expressed during the early stage (0–3W) and was identified as a hub gene in the brown module. In contrast, *COL11* was upregulated during the middle to late stages (9–15W), while *COL16* showed high expression in both early (0–3W) and late (15W) stages (Figure 3). This temporal expression signature indicates that leaves continuously perceive and integrate photoperiod signals throughout floral bud morphogenesis. It is possible that different *COL* members execute distinct regulatory functions at different developmental phases, as has been suggested in previous studies [40]. *AP2* genes play important roles in floral organ development [41]. In this study, four *AP2* genes were highly expressed during the mid-stage (9–12W; Figure 3), coinciding with the critical period for blueberry floral organ primordium differentiation. Their expression pattern and their role as hub genes in the red module highlight the importance of *AP2* during floral bud morphogenesis in blueberry.

The synergistic action of hormone signaling networks appears to be a key driver facilitating the functional transition of leaves and supporting floral bud differentiation. In this study, a large cohort of genes involved in signal transduction and biosynthesis/metabolism across multiple hormone pathways exhibited concentrated high expression during the middle to late stages (post-9W) of floral bud morphogenesis, indicating highly active hormonal networks within leaves during this period (Figure 4). The brassinosteroid signaling pathway contained the largest number of genes (101), displaying high abundance within the blue and turquoise modules (genes including *BRI1*, *BSK*, *BAK1*, *BIN2*, and *XTH23*), with most highly expressed from 9–15W, spanning the middle to late morphogenesis phases. As multifunctional regulators of plant reproductive development, BRs promote floral formation and proper floral organ morphogenesis by modulating cell expansion and division and interacting with other hormonal pathways [42]. Their sustained transcriptional activity in blueberry leaves during this period may be linked to the rapid morphogenesis of floral organs, although direct evidence for this link in blueberry is still needed. Ethylene biosynthesis and metabolism involved the largest number of genes (51). Previous studies have reported the involvement of ethylene and its related metabolic genes in floral induction and floral organ development across various plant species [43]. Furthermore, the key ethylene signaling transcription factor *ERF1* was identified as a hub gene in the turquoise module. While *ERF1* is known to play roles in stress responses and floral organ development in model species [44], its specific function in blueberry floral bud morphogenesis remains to be investigated. Jasmonic acid signaling interacts with key flowering genes like *FT* and *CO* to influence flowering time and plays a positive role in floral organ development, particularly in stamen and anther development [45]. In our study, numerous genes related to JA signal transduction (*MYC2*, *TIFYs*) and biosynthesis (*LOXs*, *AOC*) were highly expressed within the blue module. Notably, the hub gene *LOX3-1* in this module participates in the initial step of jasmonic acid biosynthesis, suggesting a potential role in modulating endogenous JA levels during floral bud morphogenesis.

Co-expression network analysis further elucidated the characteristic hormone synergy at different stages (Figure 6). The hub genes in the brown module (early stage) included *COL9*, *ARR12*, *PLA2*, and a transcription factor MYB-related. *COL9* may be involved in photoperiod signal perception; *ARR12* is a positive regulator of cytokinin signaling [46]; *PLA2* is involved in JA biosynthesis [45]; and MYB-related transcription factors are broadly involved in plant secondary metabolism and developmental regulation [47]. The co-expression of these genes in early-stage leaves suggests a possible role in integrating light and cytokinin signals to maintain basic leaf functions, perceive environmental changes, and initiate floral differentiation. Hub genes in the red module (mid-stage), including *AP2*, *DELLA*, *BCH*, *HSF,* are closely associated with regulating the functional transition of leaves. *AP2* is involved in regulating the shift towards floral organ development [41]; *DELLA*, a negative regulator of GA signaling, may coordinate floral primordium initiation and organ development [48]; *BCH* participates in carotenoid metabolism, thereby influencing ABA biosynthesis [49]; and *HSF* is an important transcription factor in the heat shock response. The activation of this module could be associated with responding to autumnal temperature changes and ongoing floral primordium differentiation, though this interpretation remains speculative. The hub genes in the blue module (mid-stage) were *SR160*, *LOX3-1*, and the MYB-related transcription factor. *SR160*, a homolog of *BRI1*, encodes a brassinosteroid receptor kinase whose high expression can enhance BR signal response, promoting cell elongation and reproductive organ development [50]. The presence of *LOX3-1* alongside JA signaling genes in the blue module raises the possibility that JA-mediated processes contribute to floral primordia differentiation. Finally, the hub genes in the turquoise module (late stage) included the flowering gene *FT*, *ERF1*, *BCAT2*, and *HB-BELL*. *FT* integrates floral signals and may sustain its function during late morphogenesis; *ERF1* integrates ethylene signals to regulate floral organ development [44]; *BCAT2* may function during floral organ development [51]; and the *HB-BELL* transcription factor family plays important regulatory roles in meristem activity and floral organ development [52]. This module may represent a core regulatory network for signal output from leaves during the terminal phase, potentially supporting bud swelling and the final differentiation of floral organs. However, direct evidence for these proposed functions in blueberry awaits future investigation through functional validation.

## 5. Conclusions

This study presents a comprehensive time-series transcriptome analysis of leaves during the critical floral bud morphogenesis phase in rabbiteye blueberry. Our findings reveal a phased and dynamic reprogramming of the leaf transcriptome, transitioning from a photosynthetic role to a hub for signal integration and metabolic support. Through WGCNA, four core modules (brown, red, blue, and turquoise) were identified, exhibiting significant associations with specific phases of floral bud morphogenesis. Key flowering-related genes, including *COL9*, *AP2*, and the florigen gene *FT*, displayed distinct temporal expression patterns, with *FT* specifically upregulated during the middle to late stages. Furthermore, complex co-expression networks involving multiple hormone pathways (notably BR, ETH, JA, and GA) and diverse transcription factor families were constructed. Hub genes such as *COL9*, *AP2*, *SR160*, *LOX3-1*, *FT*, *ERF1,* and *MYB-related* transcription factor gene were identified within these networks, suggesting their central roles in integrating environmental and hormonal signals in leaves to regulate the floral bud morphogenesis process. These findings advance our understanding of reproductive growth regulation in woody perennials and provide valuable genetic resources for blueberry breeding and cropping season management. However, as the candidate genes proposed in this study are primarily based on co-expression analysis, future research should focus on functional validation of these genes through approaches such as overexpression or silencing.

## Figures and Tables

**Table 1:** Sequencing data quality and mapping statistics.

Sample	Clean Data	Clean Reads	Mapped Reads	Sample
L-0W-1	7,400,575,256	49,830,272	44,695,777	89.70%
L-0W-2	6,221,723,935	41,835,584	37,538,949	89.73%
L-0W-3	5,940,480,492	39,995,986	35,978,380	89.95%
L-3W-1	6,697,807,935	44,911,748	40,364,980	89.88%
L-3W-2	6,162,066,985	41,367,318	37,238,747	90.02%
L-3W-3	6,300,232,431	42,361,024	38,107,694	89.96%
L-6W-1	6,938,676,972	46,604,036	42,016,262	90.16%
L-6W-2	5,947,083,684	40,055,158	36,081,049	90.08%
L-6W-3	8,416,013,982	56,770,588	50,510,127	88.97%
L-9W-1	6,293,537,059	42,248,518	37,879,510	89.66%
L-9W-2	6,771,851,521	45,521,350	40,808,379	89.65%
L-9W-3	5,909,014,939	39,663,252	35,363,165	89.16%
L-12W-1	6,344,407,840	42,513,334	36,072,611	84.85%
L-12W-2	6,324,665,702	42,395,798	37,834,162	89.24%
L-12W-3	6,198,097,014	41,607,592	36,995,235	88.91%
L-15W-1	7,367,360,870	49,604,640	44,608,579	89.93%
L-15W-2	8,217,757,515	55,566,726	49,734,772	89.50%
L-15W-3	8,227,875,482	55,507,340	49,798,067	89.71%

Samples listed in the first column of the table are leaf samples collected on the day of sampling initiation (0W), and at 3 weeks (3W), 6 weeks (6W), 9 weeks (9W), 12 weeks (12W), and 15 weeks (15W) thereafter, covering the six time points from the cessation of shoot growth to bud swelling in blueberry. The Arabic numerals 1, 2 and 3 denote replicates.

## Data Availability

The raw transcriptomic data have been deposited in the China National Genomics Data Center (https://ngdc.cncb.ac.cn/) under BioProject accession number PRJCA056783.

## Supplementary Materials

The following supporting information can be downloaded at: https://www.mdpi.com/article/10.3390/genes17030317/s1, Figure S1, Correlation Heatmap and PCA Clustering Analysis Diagram of the 18 RNA-Seq libraries; Table S1, Primer sequences for qPCR.
