# Supplementary figures and images for: Transcriptomic Reprogramming in Leaves During Floral Bud Morphogenesis in Blueberry
Source: Genes (Basel). 2026 Mar 14;17(3):317. doi: 10.3390/genes17030317 (PMC13025858; doi:10.3390/genes17030317)

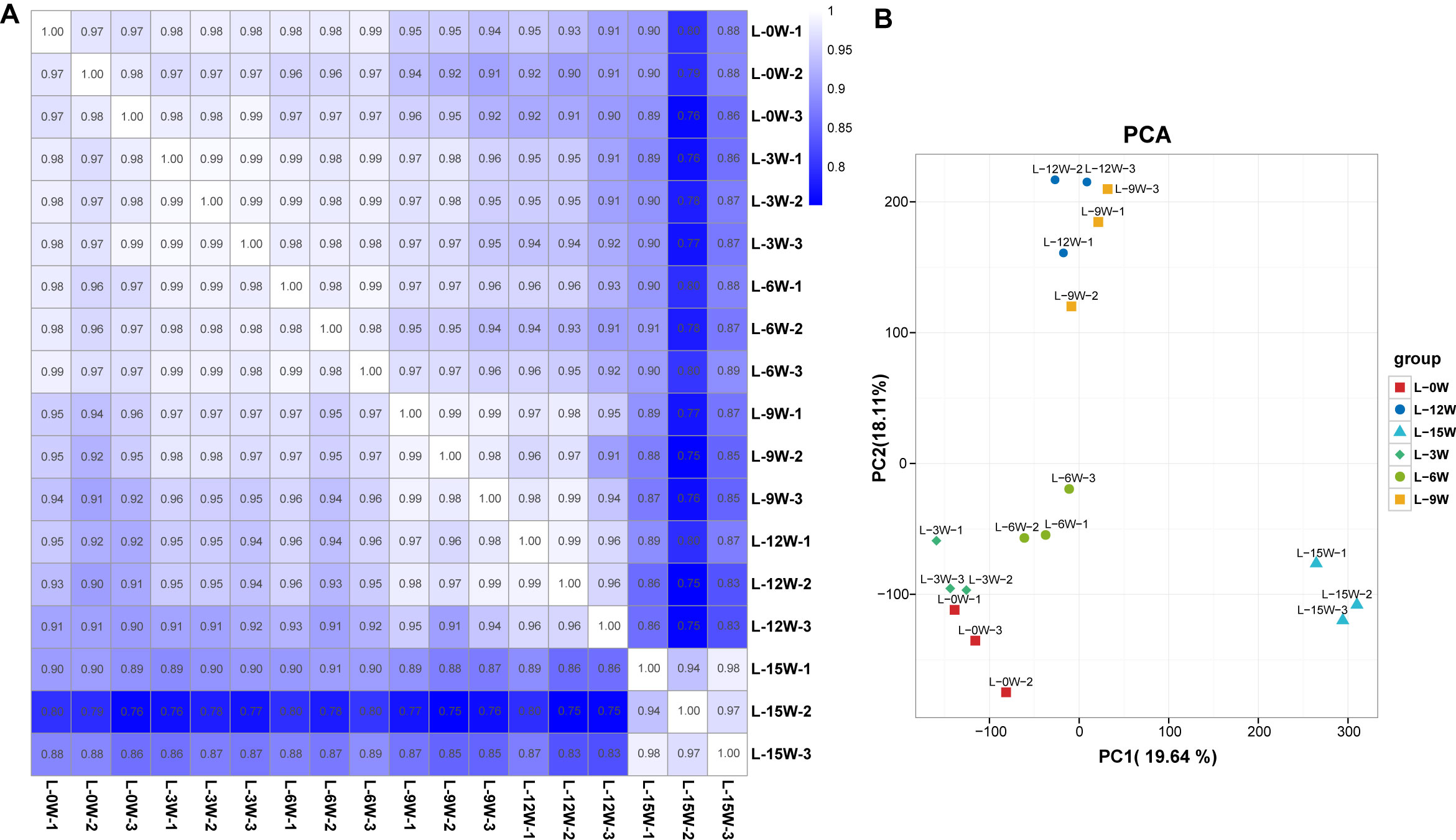

Supplement: Supplementary file 1 [file genes-17-00317-s001.zip › Figure S1. Correlation Heatmap and PCA Clustering Analysis Diagram of the 18 RNA-Seq libraries.jpg]
